# Relationship between bone mineral density and strength derived from 3D-shaper and HR-pQCT in patients with X-linked osteoporosis related to *PLS3*

**DOI:** 10.1007/s11657-026-01656-2

**Published:** 2026-01-21

**Authors:** Z. Zervou, R. Mabrouk, E. F. S. van Velsen, M. S. A. M. Bevers, E. Alizadeh, J. P. van den Bergh, M. C. Zillikens

**Affiliations:** 1 Erasmus MC Bone Center, Department of Internal Medicine, Erasmus Medical Center, Rotterdam, the Netherlands; 2 Department of Internal Medicine, VieCuri Medical Center, Venlo, the Netherlands; 3 NUTRIM Institute of Nutrition and Translational Research in Metabolism, Maastricht University Medical Center, Maastricht, the Netherlands; 4 Department of Biomedical Engineering, Eindhoven University of Technology, Eindhoven, the Netherlands; 5 Universitat Pompeu Fabra, Barcelona, Spain; 6 Department of Internal Medicine, Subdivision of Rheumatology, Maastricht University Medical Center, Maastricht, the Netherlands; 7 Rg-5, Erasmus University Medical Center, Dr. Molewaterplein 40, 3015 GD Rotterdam, the Netherlands

**Keywords:** X-linked osteoporosis, PLS3, 3D-Shaper, HR-pQCT

## Abstract

***Summary*:**

In individuals with *PLS3* genetic variants, HR-pQCT and 3D-DXA analysis indicate a larger trabecular than cortical deficit. This is consistent with the lower aBMD from DXA at the lumbar spine than at the total hip.

**Introduction:**

X-linked osteoporosis due to *PLS3* genetic variants is a rare disease, clinically affecting men more than women. This study aimed to explore relationships between parameters obtained from DXA, high-resolution peripheral quantitative computed tomography (HR-pQCT), and 3D-DXA in thirteen adults with *PLS3* variants.

**Methods:**

The parameters were for 3D-DXA: trabecular volumetric bone mineral density (Tb.BMD), cortical surface BMD (Ct.sBMD), and bone strength at the total hip using 3D-DXA-based finite element models (3D Shaper® software, v2.14.0), and for HR-pQCT: Tb.BMD, cortical volumetric BMD (Ct.BMD), and bone strength at the distal radius and tibia using micro-finite element analysis. Results were compared with normative data of the scanner system (DXA) or from literature (3D-DXA, HR-pQCT) and expressed as median *Z*-scores. Spearman correlations were determined between the different parameters.

**Results:**

aBMD at the lumbar spine was numerically lower (median *Z*-score − 1.7) than at the total hip (*Z* −1.3). 3D-DXA revealed a low Tb.BMD (*Z* −1.9), while Ct.sBMD was less affected (*Z* −1.0). HR-pQCT also showed a lower Tb.BMD (*Z* −1.9 at the radius and −2.5 at the tibia) than Ct.BMD (*Z *−0.4 and −1.3, respectively). 3D-DXA parameters were highly correlated with each other (*p* < 0.001) and with aBMD. No significant correlations were found between any of the 3D-DXA and HR-pQCT parameters.

**Conclusions:**

Both HR-pQCT and 3D-DXA showed lower trabecular than cortical BMD in individuals with *PLS3* variants, in line with the lower aBMD at the lumbar spine than at the total hip. Studies in larger cohorts and other bone disorders are needed to examine relationships between imaging modalities and their ability to predict bone fragility.

**Supplementary Information:**

The online version contains supplementary material available at 10.1007/s11657-026-01656-2.

## Introduction

Osteoporosis is a common age-related disorder characterized by a decreased bone mass and microarchitectural deterioration and a consequent increased susceptibility to fractures [1]. Different genetic variants, e.g., in *COL1A1 *and* COL1A2*, can lead to rare monogenic forms of osteoporosis such as osteogenesis imperfecta [2], which is the most common monogenetic form of increased bone fragility [3]. Another gene that is linked to early-onset osteoporosis is *PLS3* [4]. When altered, it can cause an X-chromosomal type of osteoporosis, thereby affecting men and boys more severely than heterozygous women [4]. Several studies described a severe and progressive osteoporosis phenotype in men, characterized by low bone mineral density (BMD), low-impact peripheral fractures starting in childhood and adolescence, and vertebral compression fractures causing severe pain and physical disability [5–7]. Heterozygous women showed a milder phenotype with a large variation in BMD and fracture incidence [5–8].

BMD is a main determinant of fracture risk [9]. The current gold standard imaging technique to determine BMD is dual-energy X-ray absorptiometry (DXA). DXA provides a two-dimensional (2D-) image and enables measurement of areal BMD (aBMD) [10], but it cannot discriminate between cortical and trabecular bone. Correspondingly, it does not provide a complete impression of bone quality and can furthermore be influenced by, e.g., degenerative changes [10]. It has been shown that aBMD has high specificity but low sensitivity for fractures [11]. Furthermore, DXA’s ability to predict fracture risk may be different in certain populations compared to postmenopausal women, i.e., in young individuals, those with diabetes mellitus, and patients with glucocorticoid-induced osteoporosis [12, 13]. High-resolution peripheral quantitative computed tomography (HR-pQCT) provides a three-dimensional (3D-) quantification of trabecular and cortical BMD and bone microarchitecture and enables estimation of bone strength [14]. Disadvantages of HR-pQCT include, however, higher financial costs and lower clinical availability than DXA and measurement of only the extremities. To alleviate these disadvantages and enable estimation of 3D cortical and trabecular bone properties on DXA, software has been developed that creates 3D-images from 2D-DXA images of the hip using a statistical shape and density model from QCT data to then enable evaluation of 3D cortical and trabecular BMD (3D-Shaper® software). These measurements have been found to correlate well with volumetric cortical and trabecular BMD from QCT [15]. Moreover, Qasim et al. [16] reported a high correlation between femur strength obtained from the 3D-DXA-based finite element (FE) models and those derived from QCT-based FE models.

A recent study from our group showed that adult men with *PLS3* genetic variants had low cortical and trabecular BMD, severely impaired cortical and trabecular microarchitecture, and low bone strength as assessed using HR-pQCT [17]. Results in three women indicated comparable abnormalities, but less pronounced than in men. So far, it is not clear whether 3D-DXA can detect similar abnormalities in the two bone compartments as HR-pQCT, in patients with rare monogenic bone diseases. Therefore, our aim was to study relationships between the parameters obtained from the different techniques. For this purpose, we assessed bone characteristics using 3D-DXA derived analysis of the total hip, which included trabecular BMD (Tb.BMD), cortical surface BMD (Ct.sBMD), and bone strength through 3D-DXA-based finite element (FE) analysis. Additionally, we obtained aBMD of the spine and total hip from DXA and trabecular volumetric BMD (Tb.BMD), cortical volumetric BMD (Ct.BMD), and bone strength from HR-pQCT-based micro-FE (μFE-) models at the distal radius and tibia, in patients with X-linked osteoporosis due to *PLS3* genetic variants.

## Subjects and methods

### Study design and participants

This single-center retrospective observational study was conducted at the Erasmus MC Bone Center, Rotterdam, The Netherlands. In order to be eligible to participate in this study, participants ≥ 18 years old, with a *PLS3* gene variant, must have had a DXA scan, HR-pQCT scan, and 3D Shaper® analysis based on the DXA scan, before the 31 st of October 2024. Participants with a *PLS3* variant were diagnosed with osteoporosis based on low *T*-scores or *Z*-scores and/or a history of fractures. Some individuals were identified through family-based genetic screening. The time between DXA and HR-pQCT acquisitions was always shorter than one year. This study was approved by the Medical Ethics Review Committee and the participants signed an informed consent form (MEC-2020-0737).

### Demographical, clinical, and imaging data collection

Epidemiological and anthropometric variables including sex, age (in years), height (in meters), weight (in kg), and body mass index (BMI, in kg/m^2^) were obtained from electronic patient records. Moreover, the total number of (non-) vertebral fractures was evaluated. Prevalent vertebral fractures were classified according to Genant et al. (grade 1, 20–25%; grade 2, 25%–40%; grade 3,  ≥ 40%) [18, 19].

aBMD measurements were obtained from DXA scans, taken with GE Lunar Prodigy Advance equipment (H8950AN enCORE™, Version 17 SP4). aBMD of the total hip (TH) and lumbar spine (L2–L4) were calculated with corresponding *Z*-scores using equipment-specific and age- and sex-adjusted reference data. For the 3D-DXA measurements, 3D Shaper® software v2.14 was used. The absolute values of Ct.sBMD and Tb.BMD at the TH were quantified, and their corresponding *Z*-scores were determined using age-and sex-matched control data from the literature [20]. Moreover, the 3D-DXA-based nonlinear FE analysis as previously described [16] was utilized to assess the strength of the femur under sideways fall onto the greater trochanter. In brief, 3D-DXA-based FE models were generated using the 3D shape and bone density models obtained by 3D-Shaper® software [15]. Cadaveric test conditions [21] were simulated, i.e., shaft end restricted and greater trochanter surface fixed in the fall direction. Subject-specific FE meshes were generated by morphing a generic 3D hexahedral mesh onto 3D surfaces reconstructed using 3D-Shaper® statistical modeling. Bone density values were mapped onto the FE mesh and converted into element-specific Young’s moduli using the density-elasticity relationships [16]. A rigid-body constraint imposed a displacement to the femur head through a reference point. Simulated failure was monitored through the nonlinear elastic-plastic force-displacement response of the model, being identified at the increment prior to curve decrease, and with the bone strength then defined as the maximum reaction force in the force-displacement curve. HR-pQCT measurements at the non-dominant distal radius and ipsilateral distal tibia were performed at VieCuri Medical Center, Venlo, The Netherlands, using second-generation HR-pQCT (XtremeCT II, Scanco Medical AG, Bruttisellen, Switzerland) [22, 23]. Analysis of the HR-pQCT scans is described in our previous study [17]. For the current study, only Tb.BMD, Ct.BMD, and bone failure load were used. With regard to bone failure load, the μFE analysis was conducted to estimate the failure load under compression. The μFE models were created by converting bone voxels, classified through filtering and segmentation, into equally sized μFE elements. An axial compression of 1% strain was applied to the models. The failure load was then estimated based on Pistoia’s criterion, which defines failure as the condition where 2% of bone voxels exceed a 0.7% strain threshold [17]. Corresponding *Z*-scores of these parameters were determined by comparison with normative data from the literature [24].

### Statistical analysis

For continuous variables, medians with interquartile ranges (IQR) rather than means and standard deviations were calculated due to the small sample size. *Z*-scores were interpreted as follows: normal (*Z*-score within the range of ± 1), low-normal (*Z*-score of −1 to −2), low (*Z*-score ≤ −2). Spearman correlations were used to determine correlations between the parameters from the different imaging modalities and analyses. Statistical significance was set at p < 0.05 (two-tailed). The correlations were not corrected for multiple testing due to the small number of patients resulting from the rarity of the disease. All analyses were performed using IBM SPSS Statistics software (version 28.0).

## Results

### Demographic, clinical, and imaging data

In total, 13 individuals (11 men, 2 women) were included. Median age was 48 (IQR 24.5–59.0) years, median height 1.77 (IQR 1.73–1.82) m, and median BMI 25.1 (IQR 21.8–26.3) kg/m^2^. Both female participants in the cohort are postmenopausal. One has been diagnosed with osteoporosis and has experienced three peripheral fractures since childhood. The other woman had no fractures. Regarding comorbidities, one male participant was found to have hypercalciuria. The proportion of vertebral and non-vertebral fractures in the cohort was 76.9% and 69.2%, respectively (Table 1). Vertebral fractures were not observed in any of the women. Regarding the results of the different imaging techniques, patients demonstrated lower *Z*-scores for trabecular bone parameters than cortical bone parameters, from both 3D-DXA and HR-pQCT (Table 2). Results did not change substantially when analyzing men only (Supplementary Table 1).

**Table 1:** Demographic and clinical characteristics among men with *PLS3* variants

*N* = 13	
Sex (men, %)	11 (84.6)
Age (years)	48.0 (24.5–59.0)
Height (m)	1.77 (1.73–1.82)
BMI (kg/m^2^)	25.1 (21.8–26.3)
Vertebral fractures (%)	10.0 (76.9)
Non-vertebral fractures (%)	9.0 (69.2)

Data are presented as median with interquartile range or number (percentage)

*BMI* body mass index

**Table 2:** Areal BMD evaluated by DXA and trabecular and cortical BMD evaluated by 3D-DXA and HR-pQCT and their corresponding *Z*-scores

*N* = 13	Median (*)	Median *z*-score (*)
DXA		
aBMD L2–L4 (g/cm^2^)	1.01 (0.93 −1.11)	− 1.7 (−2.3; −1.1)
aBMD TH (g/cm^2^)	0.83 (0.78–0.93)	− 1.3 (−2.0; −0.7)
3D-DXA total hip		
Tb.BMD (mg/cm^3^)	129.0 (109.0; 148.5)	−1.9 (−2.9; −1.3)
Ct.sBMD (mg/cm^2^)	145.0 (131.5; 157.0)	−1.0 (−2.0; −0.5)
HR-pQCT radius		
Tb.BMD (mg HA/cm^3^)	111.4 (79.5; 124.6)	−2.5 (−3.8; −1.6)
Ct.BMD (mg HA/cm^3^)	823.9 (788.8; 881.7)	−1.3 (−3.6; −0.9)
HR-pQCT tibia		
Tb.BMD (mg HA/cm^3^)	133.4 (113.1; 147.9)	−1.9 (−2.6; −1.8)
Ct.BMD (mg HA/cm^3^)	873.7 (754.4; 922.5)	−0.4 (−2.6; 0.3)

Data are presented as median with interquartile range (IQR)

*TH* total hip, *aBMD* areal bone mineral density, *Tb.BMD* trabecular volumetric bone mineral density, *Ct.sBMD* cortical surface bone mineral density, *Ct.BMD* cortical volumetric bone mineral density

### Correlation

**Fig. 1:**
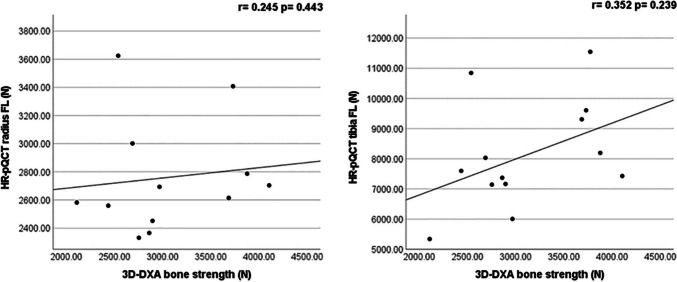
Relationship between bone strength measured by 3D-DXA-based FE models at the total hip and by HR-pQCT-based μFE at the radius (left) and tibia (right). Bone strength measured at the distal radius and tibia using HR-pQCT and at the total hip using 3D-DXA. No significant correlations were found between bone strength obtained from each technique. *r* represents the correlation coefficient, and *p*-value refers to the outcome of the Spearman correlation test. FL, failure load

Spearman correlations between the absolute values of the parameters from 3D-DXA, DXA, and HR-pQCT are shown in Table 3. 3D-DXA parameters (Tb.BMD and Ct.sBMD) were highly correlated with each other (*r* = 0.798, *p *< 0.001) and with aBMD. More specifically, Tb.BMD and Ct.sBMD by 3D-DXA were highly correlated with aBMD of TH (*r* = 0.841, *p* < 0.001 and *r* = 0.905, *p* < 0.00, respectively). Moreover, Tb.BMD by 3D-DXA was highly correlated with aBMD of L2–L4 (*r* = 0.753, *p* = 0.003). Tibial Tb.BMD by HR-pQCT was significantly correlated with aBMD of L2–L4 (*r* = 0.747, *p* = 0.03). There was no significant correlation between HR-pQCT parameters at radial and tibial measurement sites and aBMD at TH. Also, no significant correlations were found between 3D-DXA parameters and HR-pQCT parameters (Table 3). Spearman correlations between bone strength at TH from 3D-DXA and at the distal radius and tibia from HR-pQCT are shown in Fig. 1. No significant correlations were found.

**Table 3:** Spearman’s correlations between 3D-DXA parameters, aBMD by DXA, and HR-pQCT

			3D-DXA total hip		DXA		HR-pQCT			
			Tb.BMD (mg/cm^3^)	Ct.sBMD (mg/cm^2^)	aBMD L2–L4 (g/cm^2^)	aBMD TH (g/cm^2^)	Radial Tb.BMD (mg HA/cm^3^)	Radial Ct.BMD (mg HA/cm^3^)	Tibial Tb.BMD (mg HA/cm^3^)	Tibial Ct.BMD (mg HA/cm^3^)
3D-DXA Total hip	**Tb.BMD (mg/cm**^**3**^**)**	*r*	1.000	**0.798**	**0.753**	**0.841**	−0.077	−0.140	0.357	−0.055
		*p*-value		**0.001**	**0.003**	** < 0.001**	0.812	0.665	0.231	0.859
	**Ct.sBMD (mg/cm**^**2**^**)**	*r*	**0.798**	1.000	0.470	**0.905**	−0.049	−0.441	0.085	−0.388
		*p*-value	**0.001**		0.105	** < 0.001**	0.880	0.152	0.782	0.190
DXA	**aBMD L2–L4 (g/cm**^**2**^**)**	*r*	**0.753**	0.470	1.000	0.511	0.273	0.021	**0.747**	0.319
		*p*-value	**0.003**	0.105		0.074	0.391	0.948	**0.003**	0.289
	**aBMD TH (g/cm**^**2**^**)**	*r*	**0.841**	**0.905**	0.511	1.000	0.112	−0.357	0.176	−0.330
		*p*-value	** < 0.001**	** < 0.001**	0.074		0.729	0.255	0.566	0.271
HR-pQCT	**Radial Tb.BMD (mg HA/cm**^**3**^**)**	*r*	−0.140	−0.441	0.021	−0.357	0.273	1.000	0.350	0.818
		*p*-value	0.665	0.152	0.948	0.255	0.391		0.265	0.001
	**Radial Ct.BMD (mg HA/cm**^**3**^**)**	*r*	−0.077	−0.049	0.273	0.112	1.000	0.273	0.615	0.531
		*p*-value	0.812	0.880	0.391	0.729		0.391	0.033	0.075
	**Tibial Tb.BMD (mg HA/cm**^**3**^**)**	*r*	0.357	0.085	**0.747**	0.176	0.615	0.350	1.000	0.593
		*p*-value	0.231	0.782	**0.003**	0.566	0.033	0.265		0.033
	**Tibial Ct.BMD (mg HA/cm**^**3**^**)**	*r*	−0.055	−0.388	0.319	−0.330	0.531	0.818	0.593	1.000
		*p*-value	0.859	0.190	0.289	0.271	0.075	0.001	0.033	

*aBMD* areal bone mineral density, *TH* total hip, *Tb.BMD* trabecular volumetric bone mineral density, *Ct.sBMD* cortical surface bone mineral density, *Ct.BMD* cortical volumetric bone mineral density

In bold are represented correlations with a *p*-value < 0.005

## Discussion

The aim of this study was to explore relationships of trabecular and cortical BMD and bone strength, in adults with *PLS3* variants, derived from 3D-DXA models at the total hip with parameters obtained by HR-pQCT at the distal radius and tibia. Both 3D-DXA and HR-pQCT analyses indicated that trabecular BMD was more affected than cortical BMD in individuals with *PLS3* genetic variants. This was consistent with the lower aBMD at the lumbar spine (more trabecular bone) than at the total hip (more cortical bone), which suggests a larger trabecular than cortical deficit. 3D-DXA parameters (Tb.BMD and Ct.sBMD) were highly correlated with each other (*p* < 0.001) and with aBMD from DXA. Moreover, Tb.BMD at the distal tibia was significantly correlated with aBMD at the lumbar spine. On the other hand, 3D-DXA parameters at the total hip were not significantly correlated with BMD and strength parameters at the distal radius and tibia from HR-pQCT. This finding aligns with the low correlation observed between aBMD at the total hip and HR-pQCT parameters, highlighting the influence of differences in anatomical measurement sites. Nonetheless, the small sample size constrains the interpretability of the correlation analysis results. In a large population–based study, DXA-derived 3D modeling parameters were also found to be highly correlated with aBMD, and they do not appear to offer additional benefits in assessing fracture risk beyond aBMD alone [25].

Findings from previous studies are consistent with our results in that they show large trabecular deficits in patients with *PLS3* genetic variants, mainly based on data obtained from bone biopsies [26–30]. In two case series, cortical bone has also been found to be altered, as reflected by a low cortical porosity [27, 29]. Reduced cortical porosity may represent a compensatory adaptation, as lower porosity is known to enhance mechanical integrity and reduce fracture risk—particularly in load-bearing bones such as the femur and radius [31]. Recently, we reported abnormalities in both the trabecular and cortical compartments, as assessed using HR-pQCT [17]. Although the impaired Ct.BMD in that study seems to contradict the normal median Ct.BMD *Z*-score in the current study, our previous study included a larger number of patients and was stratified by sex, with women exhibiting notably fewer abnormalities, while the sample size in the current study did not allow such stratification. The absolute results in our study on trabecular and cortical BMD differed between 3D-DXA and HR-pQCT. Specifically, the *Z*-scores of Tb.BMD at the distal radius from HR-pQCT were lower than the *Z*-scores of Tb.BMD at the hip from 3D-DXA and Ct.BMD *Z*-scores at the tibia from HR-pQCT were higher than Ct.sBMD *Z*-scores at the hip from 3D-DXA. This could be attributed to the different scan locations and any different reference databases from which the *Z*-scores were calculated for HR-pQCT and 3D-DXA.

So far, 3D-DXA has been compared with QCT in two studies, but not with HR-pQCT. A study of 157 study individuals, using DXA scans from scanners of three different manufacturers, found a significant and strong correlation between trabecular BMD from QCT and 3D-DXA (*R* = 0.86) and between cortical BMD derived from the two methods (*R* = 0.93) [15]. An ex-vivo study by Dudle et al. [28] examined integral BMD at various locations of the femur, using 3D-DXA, and compared these measurements to those obtained through QCT, also reporting a significant correlation of the BMD measurements between the two methods. In the current study, we found no significant correlations between trabecular and cortical BMD obtained from 3D-DXA at the total hip and HR-pQCT at the distal radius and tibia. Several reasons may explain this lack of significant correlations with HR-pQCT in our study and the significant correlations with QCT in previous studies. For example, the current and previous comparison between 3D-DXA and QCT was based on the same skeletal site (femur), while HR-pQCT evaluates the radius and tibia. Furthermore, 3D-DXA calculations are based on statistical shape models derived from hip QCT images and thus adequate correlation between 3D-DXA and QCT may be expected. It also indicates that 3D-DXA concerns derived measurements of volumetric BMD, while HR-pQCT directly measures BMD. Besides that, the larger cohort in the previous studies may give more power to find associations. With regard to the bone strength measurements based on 3D-DXA and HR-pQCT, additional differences in the FE analyses (e.g., boundary conditions, applied loading, material properties) may contribute to differences in outcomes. All in all, although our study shows similarities between 3D-DXA and HR-pQCT regarding impairment of BMD between the cortical and trabecular compartment, it reveals no significant correlations between the parameters of the two methods. This indicates that both techniques assess different aspects of bone characteristics in different locations and that, based on the results of the current study, 3D-DXA cannot serve as a substitute for the more advanced but less commonly accessible HR-pQCT scans. In contrast, strong correlations were found between 3D-DXA and aBMD, which may be explained by the fact that 3D-DXA values were derived from the same DXA images, and at the same anatomical site.

While DXA offers a 2D evaluation of bone density, 3D-DXA generates a 3D model of the proximal femur, facilitating a more comprehensive assessment of both cortical and trabecular bone structures. This improved analysis can assist clinicians in tracking changes in bone health over time [32]. Nevertheless, certain methodological limitations must be acknowledged. Since DXA images do not completely represent cortical bone, 3D-DXA depends on statistical modeling instead of direct measurements, which may result in less precise estimations of cortical parameters [33].

This is the first study to analyze the relationships between 3D-DXA and HR-pQCT to assess cortical and trabecular volumetric BMD in patients with *PLS3*-gene related osteoporosis. Yet, there are some limitations. Firstly, the small sample size makes it difficult to interpret the statistical results with certainty. However, in rare diseases such as *PLS3*-related osteoporosis, it is difficult to obtain large cohorts. Therefore, we think it is relevant to include the results of the correlations in our study. Moreover, as mentioned above, 3D-DXA parameters were obtained at a different skeletal location than the HR-pQCT parameters. Additionally, we did not assess the Tb.BMD in the lumbar spine, as this cannot be accomplished with either 3D-Shaper or HR-pQCT. However, such an evaluation would provide further insights into the comparison of trabecular bone between both methods since trabecular bone is largely pronounced in lumbar vertebrae.

In conclusion, both HR-pQCT and 3D-DXA analyses indicate a larger trabecular than cortical deficit. This is consistent with the lower aBMD from DXA at the lumbar spine than at the total hip. We found no significant correlations between trabecular and cortical BMD parameters and bone strength derived from 3D-DXA and HR-pQCT in patients with *PLS3* gene variants, suggesting that the two methods measure different characteristics of bone structure and strength at different skeletal sites. A high correlation was observed among 3D-DXA parameters and aBMD. These findings imply that, for patients with X-linked osteoporosis related to *PLS3*, 3D-DXA provides different information than HR-pQCT regarding trabecular and cortical BMD. However, interpretation of the correlation analysis is limited due to the small sample size. Future prospective research in larger cohorts of individuals suffering from *PLS3*-related osteoporosis and other bone diseases is needed to study the relationships between the different modalities and their association with fracture prediction, as well as to assess the added value of 3D-DXA to the current gold-standard DXA.

## Supplementary Information

Below is the link to the electronic supplementary material.

ESM 1 Supplementary Material 1 (DOCX 25.8 KB)

## Data Availability

Sharing raw or processed individualized sequencing results of the patients is not allowed due to General Data Protection Regulation (GDPR). Requests to access the datasets should be directed to MCZ, m.c.zillikens@erasmusmc.nl.
