# Supplementary material for: Relationship between bone mineral density and strength derived from 3D-shaper and HR-pQCT in patients with X-linked osteoporosis related to PLS3
Source: Arch Osteoporos. 2026 Jan 21;21(1):27. doi: 10.1007/s11657-026-01656-2 (PMC12823622; doi:10.1007/s11657-026-01656-2)
Supplement: Supplementary file 1 — Supplementary Material 1 (DOCX 25.8 KB) [file 11657_2026_1656_MOESM1_ESM.docx]

Supplementary table 1. Areal BMD evaluated by DXA, trabecular and cortical BMD evaluated by 3D- Shaper and HR-pQCT in men and their corresponding Z-scores, expressed as median values in males

| N=11 | Median (*) | Median z-score (*) |
| --- | --- | --- |
| **DXA** | | |
| aBMD L2-L4 (g/cm^2^) | 1.01 (0.91 -1.10) | - 1.7 (-2.4; - 1.3) |
| aBMD FN (g/cm^2^) | 0.82 (0.76 – 0.91) | - 1.3 (-2.3; - 0.4) |
| aBMD TH (g/cm^2^) | 0.83 (0.74 – 0.95) | - 1.4 (-2.2; - 1.0) |
| **3D-DXA Total hip** | | |
| Tb.BMD (mg/cm^3^) | 131.0 (121.0 - 149.0) | -2.0 (-3.1; -1.5) |
| Ct.sBMD (mg/cm^2^) | 146.0 (129.0 - 161.0) | -1.1 (-2.1; -0.3) |
| **HR-pQCT radius** | | |
| Tb.BMD (mg/mm^3^) | 111.4 (76.3 - 119.0) | -2.9 (-4.1; -1.9) |
| Ct.BMD (mg/mm^3^) | 797.2 (765.6 - 861.5) | -1.8 (-3.0; -0.9) |
| **HR-pQCT tibia** | | |
| Tb.BMD (mg/mm^3^) | 133.4 (111.0 - 155.7) | -2.0 (-2.8; -1.8) |
| Ct.BMD (mg/mm^3^) | 821.6 (727.3 - 919.3) | -1.2 (-3.1; 0.2) |
| *T-score  Data are presented as median with interquartile range (IQR)  aBMD: areal bone mineral density; Tb.BMD: trabecular volumetric bone mineral density, Ct.sBMD: cortical surface bone mineral density; Ct.BMD: cortical volumetric bone mineral density | | |
